# AI-2/Lux-S Quorum Sensing of *Lactobacillus plantarum* SS-128 Prolongs the Shelf Life of Shrimp (*Litopenaeus vannamei)*: From Myofibril Simulation to Practical Application

**DOI:** 10.3390/foods11152273

**Published:** 2022-07-29

**Authors:** Yuan Li, Taige Liu, Xianghong Meng, Yilin Qian, Shijie Yan, Zunying Liu

**Affiliations:** 1 College of Food Science and Engineering, Ocean University of China, Qingdao 266003, China; liyuan19930603@outlook.com (Y.L.); mengxh@ouc.edu.cn (X.M.); qianyl92@outlook.com (Y.Q.); 2 College of Food Science and Biological Engineering, Tianjin Agricultural University, Tianjin 300392, China; tiger980504@163.com

**Keywords:** AI-2/Lux-S quorum sensing, *Lactobacillus plantarum* SS-128, myofibrils

## Abstract

Retarding the protein deterioration of shrimp during storage is important for maintaining its quality. *Lactobacillus plantarum* SS-128 (*L. plantarum* SS-128) is a biocontrol bacterium that can effectively maintain the fresh quality of food. This research establishes a myofibril simulation system and refrigerated control system to explore the impact of *L. plantarum* SS-128 on the quality and shelf life of refrigerated shrimp (*Litopenaeus vannamei*). Through the bacterial growth assay and AI-2 signal molecule measurement, the effect of the AI-2/LuxS quorum sensing (QS) system of *L. plantarum* SS-128 and shrimp spoilage bacteria was established. In the myofibril simulation system, a study on protein degradation (dimer tyrosine content, protein solubility, sulfhydryl content, and carbonyl content) showed that adding *L. plantarum* SS-128 effectively slowed protein degradation by inhibiting the growth of food pathogens. The application to refrigerated shrimp indicated that the total volatile basic nitrogen (TVB-N) value increased more slowly in the group with added *L. plantarum* SS-128, representing better quality. The total viable count (TVC) and pH results exhibited similar trends. This study provides theoretical support for the application of *L. plantarum* SS-128 in storing aquatic products.

## 1. Introduction

*Litopenaeus vannamei* is a representative shrimp at the forefront of global aquaculture production and consumption. It contains a variety of proteins and amino acids that the human body needs. It is a raw food material with a good flavor and nutrition. In 2020, the culture volume of *Litopenaeus vannamei* in China was 1,197,735 tons, accounting for more than 80% of the total shrimp culture volume. However, approximately one-third of *Litopenaeus vannamei* is wasted before consumption [1]. With the improvement in people’s living standards, the demand for *Litopenaeus vannamei* has increased, and the requirements for the edible quality of *Litopenaeus vannamei* have further improved. The products that fail to maintain a good edible quality during refrigeration suffer potential waste risks. In 2021, the overall catch of the crustacean aquatic products represented by shrimp decreased by 8.37%, and the gap between supply and demand continued to expand. Therefore, it is an urgent problem to explore a refrigeration regulation system to effectively maintain the edible quality of shrimp.

Biocontrol lactic acid bacteria are lactic acid bacteria with biocontrol functions. They have attracted extensive attention due to their advantages, including pollution-free and special biocontrol effects. Studies have shown that the biocontrol lactic acid bacteria can protect food quality. Inoculating a lactic acid bacterial solution onto fresh beef slices could significantly inhibit the growth of *Listeria* and *Staphylococcus* aureus [2]. In addition, the preservation of biocontrol lactic acid bacteria is often used in smoked meats and other meat products [3]. Adesokan et al. sprayed *Lactobacillus plantarum* fermentation broth onto smoked meat products, significantly inhibiting the harmful microorganism proteus growth [4]. Castellano et al. sprayed *Lactobacillus curvatus* onto meat slices, which can prolong the cold storage shelf life to 36 days, and effectively inhibited the growth of *Listeria* and *Cyclomyces thermodead* [5].

The quorum sensing (QS) system of biocontrol lactic acid bacteria is a crucial method for food preservation. QS is a communication phenomenon between bacteria. Bacteria regulate their physiological behavior according to population density through this system. Bacteria can produce signal molecules and sense the molecules produced by other bacteria to respond to the bacteria co-existing in the environment. When the number of signaling molecules reaches a certain threshold, the relevant genes in the bacteria begin to regulate bacterial population behavior and intercellular communication to adapt to the environmental changes. The manipulation of the QS systems has been widely used in pharmaceuticals, clinical research, agriculture, ecological applications, biological control, and food preservation.

The AI-2/LuxS QS system is essential for the biocontrol of the lactic acid bacteria. Biocontrol lactic acid bacteria can sense competitive environmental pressure through the AI-2/LuxS QS system and play an essential role in the interspecific and intraspecific information exchange of bacteria, including *Shewanella*, an important spoilage bacterium during the storage of aquatic products [6]. In the QS system of AI-2/LuxS, the *LuxS* gene mediates the signal transduction of AI-2 and controls the different behaviors of the lactic acid bacteria. The biocontrol of lactic acid bacteria can control the production of bacteriocin through the AI-2/LuxS QS system, inhibit competitors, and promote their growth. The studies have shown that the AI-2/LuxS QS system of lactic acid bacteria significantly impacts on the production of bacteriocin [7]. When Lactobacillus nc8 was cocultured with Enterococcus faecium 6t1a-20, the *Lactobacillus* nc8 inhibited the growth and metabolism of the latter [8].

Protein is a significant component in shrimp muscle, and myofibrils are proteins that make up the myofibrils in muscle, including tropomyosin, myosin, and actin. Myofibrils occupy approximately 55–60% of the proteins in aquatic products. It has been shown that the degree of protein is correlated with the spoilage status of the aquatic products [9,10]. Research on myofibrillar changes during the refrigerated storage of muscle foods could reflect the status of freshness. Currently, research on the cold storage of food by biocontrol lactic acid bacteria focuses on extending the shelf life [11,12,13], and there are few studies on the protective effect of the texture and other edible qualities. Li et al. found that biocontrol lactic acid bacteria lab-1 can reduce the texture deterioration in *Litopenaeus vannamei* during cold storage, but the mechanism of delaying the quality deterioration has not been studied [14]. Establishing a scientific control system of biocontrol lactic acid bacteria in cold storage can effectively improve the damage caused by quality deterioration in the process of cold storage of *Litopenaeus vannamei*, to reduce the waste of aquatic resources. A simulated regulation system of “biocontrol lactic acid bacteria-*Litopenaeus vannamei*” was established to explore the regulation of biocontrol lactic acid bacteria on the protein degradation of the refrigerated shrimp in multiple dimensions through microbial growth kinetics and biochemistry. The regulation system of “biocontrol lactic acid bacteria-*Litopenaeus vannamei*” during refrigerated storage was established to explore the effect and mechanism of biocontrol lactic acid bacteria on the protein degradation and the quality deterioration of refrigerated shrimp through a protein comprehensive evaluation and determination of myofibril changes.

## 2. Materials and Methods

### 2.1. Materials

Shrimp (*Litopenaeus vannamei*) weighing 45 ± 5 g were purchased from the local fishery market at Qidong Rd., Qingdao, and were transported to the laboratory on ice within half an hour.

*Lactobacillus plantarum* SS-128 (*L. plantarum* SS-128) was extracted from the intestine of blackhead fish and preserved by the Laboratory of Aquatic Products Higher Application Technology, Ocean University of China. The *luxS*-mutant strain of *Lactobacillus plantarum* SS-128 (*L. plantarum ΔluxS*) was constructed previously in the lab. *Shewanella baltica* A02 (*S. baltica* A02) was isolated from spoiled shrimp. Pro donated the 4,5-dihydroxy-2,3-pentanedione (DPD),Ming Li’s lab in the College of Pharmacy, Ocean University of China. The MRS broth, agar, and *Lactobacillus* medium were purchased from Qingdao Haibo Biotechnology Co., Ltd., China. The NaCl, N,N,N’,N’-tetramethylethylenediamine, Na_2_HPO_4_·12H_2_O, and other analytical pure chemical reagents were purchased from Sinopharm Group Chemical Reagent Co., Ltd., Shanghai, China.

### 2.2. Extraction of Myofibrils

The peeled and eviscerated shrimp were rinsed twice with sterile water on a clean bench. Approximately 20 g of the minced shrimp sample was added to 10 times the volume of precooled buffer A (100 mM NaCl-1 mM EDTA-20 mM PBS, in which the PBS was comprised of 15.6 mmol L^−1^ Na_2_HPO_4_ and 3.5 mmol L^−1^ KH_2_PO_4_, pH = 7.0), homogenized for 30 min, and then centrifuged at 1000× *g* (3000 rpm) at 4 °C for 15 min. The supernatant was sarcoplasmic protein. The precipitate obtained by centrifugation was centrifuged under the same conditions with buffer A at five times the volume of the original sample weight. The supernatant was discarded, five times the volume of buffer A was added to the precipitate for filtration, the gauze was washed with five times the volume of buffer A, and the filtrate was centrifuged under the same conditions to obtain the precipitate. The process of filtration and centrifugation was repeated two–three times, and finally, the precipitate was dissolved with buffer B (1 M NaCl-50 mM PBS, pH = 7.0). The dissolved solution contained myofibrils. If not dissolved well, 100–150 mL buffer B was added to the precipitate, homogenized and centrifuged, and the supernatant was collected. The obtained myofibril solution was refrigerated at 4 °C until use.

### 2.3. Bacterial Incubation and Establishment of the Myofibril Simulation System

The samples were divided into four groups with the following treatments:

(1) Group *L.* SS-128: One milliliter of 10^7^ log CFU/g *L. plantarum* SS-128 and 1 mL of 10^7^ log CFU/g *S. baltica* A02 were mixed with 100 mL of myofibril solution;
(2) Group *L.ΔluxS*: 1 mL of 10^7^ log CFU/g *L. plantarum ΔluxS* and 1 mL of 10^7^ log CFU/g *S. baltica* A02 were mixed with 100 mL of myofibril solution;
(3) Group *L.ΔluxS* + DPD: 1 mL of 10^7^ log CFU/g *L. plantarum ΔluxS*, 24 μg of DPD, and 1 mL of 10^7^ log CFU/g *S. baltica* A02 were mixed with 100 mL of myofibril solution;
(4) Group Control: 100 mL of myofibril solution.

All of the groups were stored at 4 °C.

### 2.4. Analysis of S. baltica A02 and Lactic Acid Bacteria Count

The bacterial density in the myofibril solution was determined by measuring the absorbance under 600 nm at 80 h. A total of 1 mL of the myofibril solution was used to coat different media, in which IA medium was used to determine the total number of the *S. baltica* A02 and cultured at 30 °C for 48 h; the MRS medium was used to determine the number of lactic acid bacteria and cultured at 37 °C for 48 h. The colony count was expressed as log CFU/g.

### 2.5. Analysis of AI-2 Activity and Protease

The AI-2 activity was measured according to the method of Qian et al. (2021) [15]. *Vibrio harveyi* BB170 was used as the indicator strain, and it was cultured in AB medium at 30 °C for 12 h and then diluted at a ratio of 1:5000. This medium was then added to the diluted *Vibrio harveyi* culture at a final concentration of 10% (*v*/*v*). The 96-well plates were vibrated at 30 °C and 100 rpm, and PBS was selected as the negative control. A Synergy H4 hybrid microplate reader (Bio-Tek, Winooski, VT, USA) was used to record the light production. The AI-2 activity was calculated as the difference in the bioluminescence level between the experimental group and the control group, presented as relative luminescence units (RLUs).

The protease activity of the bacteria in the myofibril simulation system was measured after 80 h of culture at 4 °C. The measurement was modified from Nicodeme et al. (2005) [16]. The bacterial solution was centrifuged at a low temperature to obtain sterile supernatant. Azocasein was used as the substrate, and the reaction system contained 200 μL 20 mg/mL azocasein and 400 μL bacterial supernatant, mixed evenly and placed in a 30 °C water bath for 1 h. Then, 600 μL trichloroacetic acid (10%, M/V) was added to precipitate the protein. In the control group, trichloroacetic acid was added first, and then the bacterial supernatant was added and centrifuged at low temperature after adding azotize to a water bath. A total of 500 μL of the supernatant was added to an equal volume of 525 mm NaOH, and the absorbance at 440 nm wavelength was expressed as the protease.

### 2.6. Protein Changes during Preservation

#### 2.6.1. Dimer Tyrosine Content

The dimer tyrosine content was measured according to the method of Li et al. (2021) [9]. The concentration of the myofibrillar protein was diluted to 2 mg/mL, and then the emission wavelength and the excitation wavelength absorbance were measured at 420 nm and 325 nm, respectively. The slit width was set as 10 nm. The value of the dimer tyrosine was expressed by the relative fluorescence value, which was calculated by dividing the absorbance value from the protein concentration with the unit of arbitrary units (A.U.).

#### 2.6.2. Protein Solubility

The protein solubility was calculated with reference to the method of Benjakul et al. (2000) [17]. The 5.0 mL myofibrillar solution was dissolved in 1.0 mol/L KCl solution, homogenized at 6000 r/min for 1 min, and then stirred in an ice bath. After centrifugation at 8000 r/min for 10 min, 5 mL of supernatant was mixed with 45 mL of 0.5 g/mL trichloroacetic acid. The mixture was centrifuged at 4 °C at 8000 r/min for 15 min, the supernatant fluid was discarded, the precipitate was washed with 0.5 g/mL trichloroacetic acid, and the combined supernatant was dissolved in 10 mL 0.5 mol/L NaOH solution. The 1.0 mL myofibrillar solution, dissolved with 10 mL 0.5 mol/L NaOH solution, was used to express the total protein content to determine the protein concentration, and the protein solubility was calculated according to Formula (1):

Protein solubility = Supernatant protein concentration/Total protein concentration(1)

#### 2.6.3. Sulfhydryl Content

The sulfhydryl content was measured with reference to the method of Eymard et al. (2009) [18]. One milliliter of myofibrillar solution at a concentration of 2 mg/mL was added to 8 mL of Tris-glycine solution (pH = 8, containing 10.4 g Tris, 6.9 g glycine, 1.2 g ethylenediaminetetraacetic acid and 8 M urea per liter). The sample was homogenized and centrifuged at 4 °C for 15 min at 8000 r/min. The precipitate was discarded, and 0.5 mL of 10 mM Ellman reagent was added to 4.5 mL of the sample. After 0.5 h of reaction at room temperature, the absorbance at 412 nm was measured. The sulfhydryl content was calculated according to the following formula:

Sulfhydryl content = (A × n)/(ε × ρ) (2)

In Formula (1), A represents the absorbance at 412 nm; n represents the dilution times; ε represents the molar absorption coefficient with a constant value of 13,600 M^−1^ cm^−1^; and ρ represents the protein mass concentration (mg/mL).

#### 2.6.4. Carbonyl Content

The carbonyl content was measured according to the method of Li et al. (2021) [9]. Two milliliters of myofibrillar solution was diluted to 5 mg/mL, and 2 mL of 10 mM 2,4-dinitrophenylhydrazine (DNPH) was added and reacted for 1 h. Then, 2 mL of trichloroacetic acid with a mass fraction of 20% was added and centrifuged at 4 °C at 8000 r/min for 15 min. The precipitate was washed three times with 2 mL of ethyl acetate and ethanol (volume ratio 1:1). A total of 5 mL of guanidine hydrochloride solution (6 M, dissolved in 20 mmol/L PBS, pH 6.5) was added and then the precipitate was dissolved in a water bath at 37 °C for 20 min. The solution was centrifuged at 8000 r/min for 5 min. The insoluble matrix was centrifuged for 5 min at 8000 r/min at 4 °C. The absorbance of the precipitate was measured at 370 nm, and the molar absorption coefficient (21,000 M^−1^ cm^−1^) was used to calculate the carbonyl content. The results were expressed as nmol/mg myofibrillar protein.

### 2.7. Measurement of Changes in Litopenaeus vannamei during Preservation

#### 2.7.1. Sample Preparation

The shrimp were peeled and eviscerated under ice anesthesia and then rinsed three times with sterile water on a clean bench. They were randomly divided into five groups. For the S. baltica A02-inoculated groups, the shrimp were immersed in 10^8^ CFU/g S. baltica A02 for 10 s and then removed for further inoculation treatment. The control group (CG) was not subjected to the inoculation treatments. Group L. SS-128 was sprayed with 10^8^ CFU/g L. plantarum SS-128 until the shrimp surface was evenly covered with the bacteria. The *L.ΔluxS* group was sprayed with L. plantarum *ΔluxS*/SS-128 at a concentration of 10^8^ CFU/g. The L. plantarum *ΔluxS* and DPD-exogenous addition group was sprayed with DPD-exogenous addition L. plantarum *ΔluxS* at a concentration of 10^8^ CFU/g.

All of the samples were packed separately in plastic bags and stored at 4 °C. The samples were removed for analysis every day.

#### 2.7.2. Total Volatile Basic Nitrogen (TVB-N)

The TVB-N value was measured according to the method of Li et al. (2019) [19]. A total of 5.0 g of minced shrimp samples was added into a 50 mL centrifuge tube with 27 mL 0.6 M HClO_4_ solution, homogenized for 30 s at 12,000 rpm in an ice bath, and then centrifuged at 4 °C at 8000 rpm for 10 min. Ten milliliters of the supernatant was placed into an automatic Kjeldahl apparatus for the TVB-N determination. The 0.05 M H_2_SO_4_ was the titration acid.

#### 2.7.3. pH Analysis

The pH values of the shrimp samples were measured, using a Testo 205 pH meter (Testo AG, Lenzkirch, Germany) by inserting an electrode into the back muscle of the shrimp.

#### 2.7.4. Lactic Acid Bacteria Count and Total Viable Count (TVC)

A total of 20 g of the minced shrimp was mixed with 180 mL of the sterile normal saline and then serially diluted (1:10) in sterile normal saline for analysis. The TVC count was performed by the pour plate method, using plate count agar and cultured at 37 °C for 48 h. The lactic acid bacteria count was performed using an IA medium and cultured at 30 °C for 48 h. The results were expressed as log_10_ CFU/g (colony forming units) per gram of shrimp.

### 2.8. 16S rRNA Analysis

The 20 g shrimp sample was minced with 80 mL sterile normal saline (0.9% *w*/*w*), fully beaten with a beating cup and filtered with four layers of gauze. Then, the filtrate was centrifuged at 10,000 rpm for 10 min at four °C. The bacterial DNA was extracted from a bacterial DNA extraction kit and detected by 0.8% agarose gel electrophoresis to observe whether the strip was clear.

Using the extracted DNA as a template, the 16S V4 hypervariable region was amplified by the primers 515f/806r. The PCR amplification procedure was as follows: 95 °C for 3 min; (95 °C for 45 s, 56 °C for 1 min, 72 °C for 90 s) × 30 cycles; then 72 °C for 10 min. The specificity of the combined PCR products was detected by electrophoresis. The samples were sent to Meiji Technology Co. and sequenced by second-generation sequencing technology. The paired-end readings were merged using FLASH, and the merged sequences were filtered using QIIME. Similar sequences were clustered into operational taxa (OTUs), based on a 97% threshold. The representative sequences selected in each OTU were annotated according to the mothur method and the taxonomic information of the Silva and SSUrRNA databases [20]. The sequencing data of the 16S rRNA sequence were stored in the database, and the login number of GenBank was No. PRJNA495736.

### 2.9. Statistical Analysis

All of the experiments were carried out in triplicate, and the mean values were calculated as the means ± SDs (*n* = 3). Duncan’s multiple range tests and analysis of variance (ANOVA) were performed in SPSS 22.0, and the images were analyzed using MATLAB 2021b.

## 3. Results and Discussion

### 3.1. S. baltica A02 Count and Lactic Acid Bacteria Count in the Myofibril Simulation System

**Figure 1:**
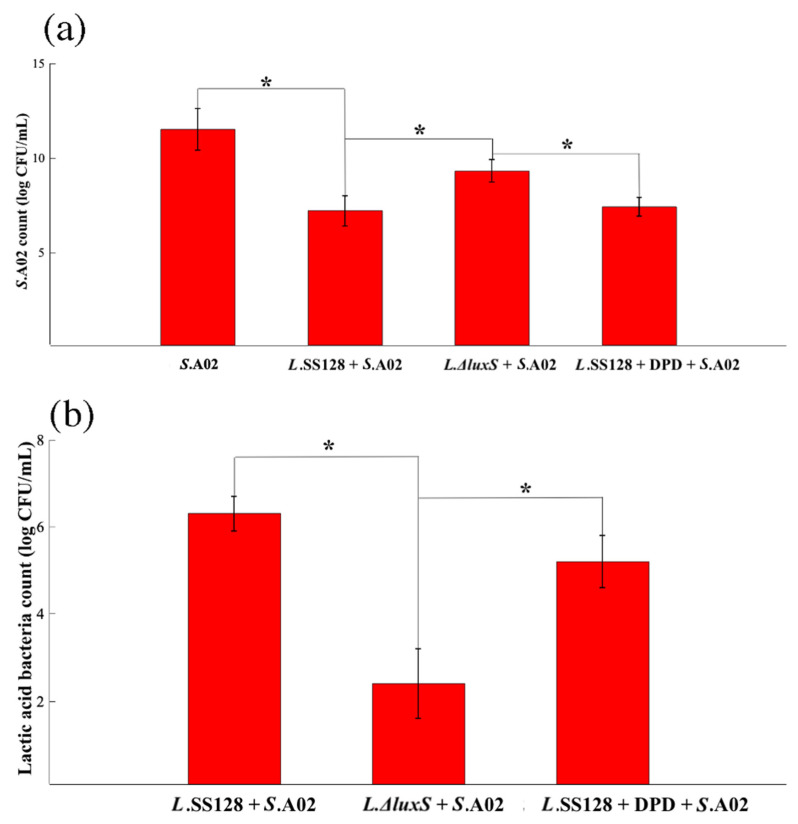
Bacteria count in the myofibril simulation system. (**a**) *S. baltica* A02 count; (**b**) lactic acid bacteria count. * Represents mean values differ significantly at *p* < 0.05.

The *S. baltica* A02 count and the lactic acid bacteria count in the myofibril simulation system at 80 h are shown in Figure 1a,b, respectively. In Group *S.* A02, no lactic acid was detected; thus, the lactic acid bacteria data of Group *S.* A02 are not shown.

The lactic acid bacteria count of the Group *L.*SS-128 + *S.* A02 was significantly higher than that of the *L*. *ΔluxS* + *S.* A02, while the *S. baltica* A02 count was significantly lower (*p* < 0.05). Li et al. reported that the AI-2/LuxS QS system in a strain of lactic acid bacteria could inhibit pathogen growth during coculture [19]. The *S. baltica* A02 count in the L. *ΔluxS* + DPD + *S*. A02 was 5.29 ± 0.63 log CFU/g, which showed no significant difference from the *L.*SS-128 + *S.*A02 group (6.24 ± 0.45 log CFU/g, *p* > 0.05). AI-2 biosynthesis is regulated by the S-adenosylmethionine (SAM) metabolic pathway. Sam produces 4,5-dihydroxy-2,3-pentanedione (DPD) under the action of methyltransferase, adenosine homocysteine nucleosidase (PFS), and s-ribosylhomocysteine lyase (LuxS). DPD is the precursor of AI-2 [21]. The lactic acid bacteria count showed similar trends, indicating that the AI-2/LuxS QS in the *L. plantarum ΔluxS* could be offset by the existence of DPD. The results of the *S. baltica* A02 count and the lactic acid bacteria count in the myofibril simulation system proved the role of the AI-2/LuxS QS system in inhibiting the growth of *S. baltica* A02, providing critical evidence for the potential spoilage bacteria-inhibiting effect in the storage of shrimp.

### 3.2. QS Index

#### 3.2.1. AI-2 Activity

**Figure 2:**
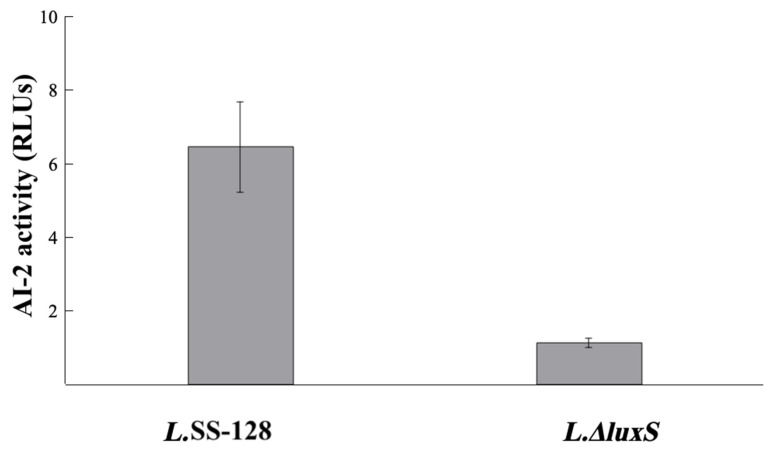
The AI-2 activity of *L.* SS-128 and *L.ΔluxS*.

The AI-2 activity of the *L. plantarum* SS-128 and *L. plantarum ΔluxS* is shown in Figure 2. The AI-2 activity of *L. plantarum* SS-128 was 6.45 ± 1.23 RLUs, significantly higher than that of *L.ΔluxS* (*p* < 0.05). Principally, the AI-2 activity should not be detected in *L. plantarum ΔluxS*. The presence of the AI-2 activity could be caused by interference from the medium. The AI-2 signaling molecule of the lactic acid bacteria is produced by the regulation of the *luxS* gene [22]. The data in Figure 1 exhibit the same results.

#### 3.2.2. Protease Activity

**Figure 3:**
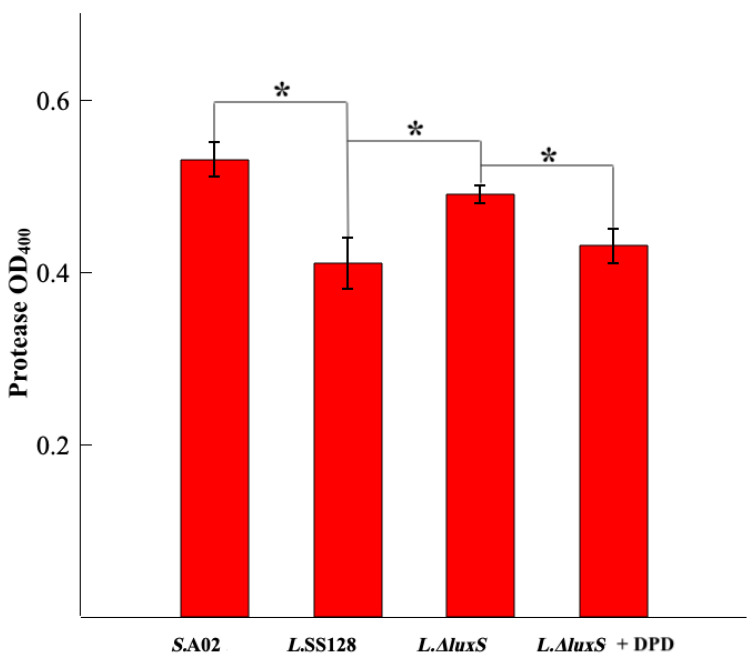
Protease content after culture for 80 h at 4 °C. * Represents mean values differ significantly at *p* < 0.05.

The effect of the AI-2/LuxS QS system of *L. plantarum* SS-128 on the protease activity of *S. baltica* A02 is shown in Figure 3. After 80 h of culture, the protease activity of the *S. baltica* A02 in Group *L.* SS-128 was 0.41 ± 0.03, which was significantly lower than that in Group *S.* A02 (*p* < 0.05). Compared with the *L*.SS-128 + *S*. A02, the protease activity of *S.* A02 in the *L.ΔluxS + S.* A02 increased significantly. Compared to the *L.ΔluxS + S*. A02, the protease activity of *S.* A02 in the *L.ΔluxS + S*. A02 + DPD decreased significantly (*p* < 0.05). During the bacterial growth, some extracellular enzymes are produced, which can decompose aquatic products rich in protein, leading to the deterioration of food [23]. Protease is an important extracellular enzyme that leads to the deterioration in the quality of aquatic products. The results showed that the AI-2/LuxS QS system of *L. plantarum* SS-128 has the potential to effectively inhibit the *S. baltica* A02 growth during preservation.

### 3.3. Protein Changes in the Myofibril Simulation System

In refrigerated aquatic products, muscle spoilage is the comprehensive effect caused by microbial activities and endogenous enzymes. The analysis of the protein changes in the myofibril simulation system of *Litopenaeus vannamei* provides theoretical evidence on the effect of *L. plantarum* SS-128 and its AI-2/LuxS QS system on the *Litopenaeus vannamei* protein.

#### 3.3.1. Dimer Tyrosine Content

**Figure 4:**
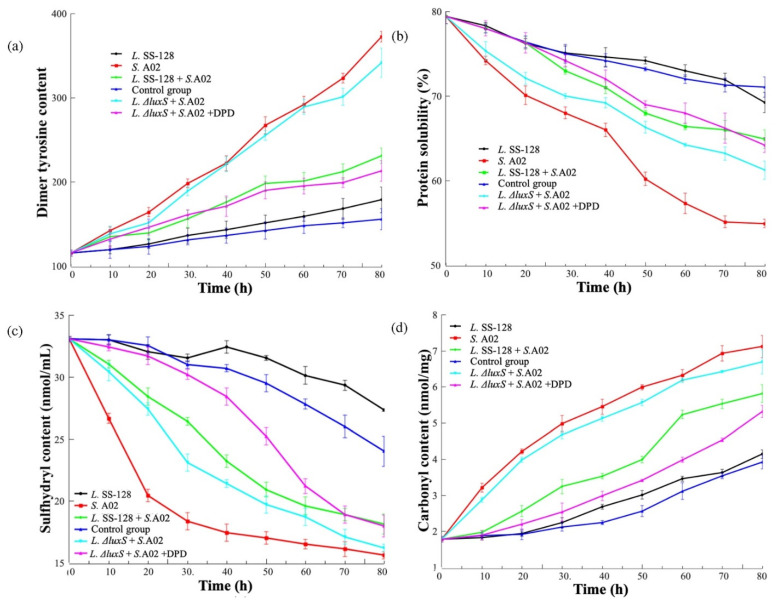
Protein changes in the myofibril simulation system. (**a**) Dimer tyrosine content; (**b**) Protein solubility; (**c**) Sulfhydryl content; (**d**) Carbonyl content.

The decline in the shrimp freshness is a complex combination of biochemical, physical, and structural changes in the proteins, lipids, and other components. During storage, the myofibrillar was hydrolyzed by spoilage microorganisms and endogenous enzymes, the tyrosine in the protein was oxidized and complexed, and the content of the dimer tyrosine increased gradually [24]. The dimer tyrosine and sulfhydryl have a strong correlation with the texture of muscle foods [25]. As shown in Figure 4a, in the myofibril simulation system, during the period of 80 h, the dimer tyrosine content of all of the groups showed increasing trends. The dimer tyrosine is an essential index indicating the degree of protein attacked by free radicals [26]. The relative fluorescence is used to express the content of the dimer tyrosine. Group *S.* A02 indicated significantly higher dimer tyrosine content than the control group (*p* < 0.05), illustrating the attack of *S. baltica* A02 on the myofibril simulation system. The dimer tyrosine content of the *L.ΔluxS* + *S.* A02 showed no significant difference from Group *S.* A02 (*p* > 0.05), while the content of the dimer tyrosine in Group *L.* SS-128 was significantly lower (*p* < 0.05). This finding indicated the critical role of the *luxS* gene in bacterial growth and metabolism in the myofibril simulation system. At 80 h, the dimer tyrosine content for Group *S.* A02, *L.ΔluxS*+*S*. A02 and the control group were 372.4 ± 6.34, 341.7 ± 17.43, and 155.4 ± 12.64, respectively. As a spoilage microorganism in shrimp, the rapid growth of *S. baltica* A02 signified the consumption of myofibrils. The dimer tyrosine content of the *L.ΔluxS* + *S.* A02+DPD and *L*. SS-128 + *S.* A02 showed no significant difference (*p* < 0.05), indicating that the AI-2/LuxS QS system in *L. plantarum* SS-128 significantly inhibited the growth of *S. baltica* A02 in the myofibril simulation system. Similar changing trends in the dimer tyrosine content in the hairtail fish myofibril were reported [9].

#### 3.3.2. Protein Solubility

The degradation of protein is not conducive to the quality of the shrimp. The protein solubility is related to the content of the hydrophobic amino acids (phenylalanine, tryptophan residues, etc.) on the surface of the protein molecules [27], and the changes in the protein solubility in the myofibril simulation system are shown in Figure 4b. The protein solubility in all of the groups decreased with the extension of time. When the spatial conformation of a protein changes, the internal hydrophobic amino acids are exposed, which changes the solubility of the protein, so the protein solubility can characterize the change in the protein conformation [28]. The protein solubility of Group *L.ΔluxS* + *S.* A02 + DPD and *L.*SS-128 + *S.* A02 at 80 h was 64.2 ± 0.91% and 64.9 ± 1.12%, respectively, significantly higher than that of the control group and the *L.*SS-128 group. Although the AI-2/LuxS QS system existing in *L. plantarum* SS-128 can inhibit the growth of *S*. A02, there is still a group of *S.* A02 that exists in the myofibril simulation system and their metabolism accelerates the decline in protein solubility by destroying the myofibrils. The protein solubility of Group *S*. A02 was lower than that of the *L.ΔluxS* + *S.* A02, which illustrates that in addition to the AI-2/LuxS QS system, there might be other pathways by which the *L. plantarum* SS-*128/L. plantarum ΔluxS* affects the growth of *S*.A02. The solubility of the myofibrillar protein isolate from hairtail (*Trichiurus lepturus*) fish is reported to decrease when exposed to external stimulation. Oxidative action could be the internal cause [29].

#### 3.3.3. Sulfhydryl Content

During the storage of shrimp, the degradation of the texture quality is obviously affected by the degradation of the protein in muscle tissue. With the progress of storage, the disulfide bond may be modified by side chains and cross-linked with other peptides [30]. In the sulfhydryl content of the myofibril simulation system (Figure 4c), the content of the protein sulfhydryl gradually decreased, indicating that the protein sulfhydryl gradually oxidized to form disulfide bonds. Hatab et al. (2022) found that the sulfhydryl content in the myofibrillar protein isolated from hairtail fish decreased after being treated with plasma, which could accelerate the conversion of the thiol group present in the protein molecules into disulfide bonds because of oxidation [29]. Corresponding to the dimer tyrosine content and protein solubility results, the addition of the *L. plantarum* SS-128 significantly inhibited the transformation from sulfhydryl to disulfide bonds in the *S.* A02-inoculated system (Group *L.*SS-128 + *S.*A02). Aggregation is the result of nonpolar interactions to take a lower energy state. The light enzymolysis region of myosin is most likely to be oxidized and then form disulfide bonds; in addition, the head region is most likely to be oxidized because the ATPase activity of myosin, that is mainly displayed by the head region, has been proved to be highly susceptible to oxidation [31]. The luxS gene participated in a critical role during the storage of the myofibril simulation system. At 80 h, the sulfhydryl content for the *L.*SS-128 + *S.* A02 was 18.12 ± 0.78 mmol/mL, which was significantly higher than that of the *L.ΔluxS* + *S.* A02 (16.2 ± 0.32 mmol/mL, *p* < 0.05).

#### 3.3.4. Carbonyl Content

Due to the combined action of the microorganisms and endogenous enzymes, MHC, α-, the degradation of actin, desmin, actin, troponin-T, and tropomyosin led to the deterioration in the shrimp quality, and the protein carbonyl increased with the increasing storage time [32]. The changes in the carbonyl content in the myofibril simulation system are shown in Figure 4d. The changes in the carbonyl content followed the same laws as the other proteins and indicates that Group *S.* A02 had the highest increasing rate of carbonyl content, and the addition of *L. plantarum* SS-128 effectively inhibited the increasing trend. The carbonyl content of the myofibril simulation system is correlated with the oxidative degree. Lee et al. reported that *L. plantarum* BCRC10357 inhibited the oxidation reaction and the spoilage of bacterial growth during the storage of tuna [33]. The inhibition of the carbonyl content indicated that the inhibition of *S. baltica* A02 growth by *L. plantarum* SS-128 retarded the oxidation reaction of the myofibril simulation system, which is evidence that the addition of *L. plantarum* SS-128 could assist with the storage of shrimp through the AI-2/LuxS QS system.

### 3.4. Changes in Litopenaeus vannamei during Preservation

#### 3.4.1. TVB-N

**Figure 5:**
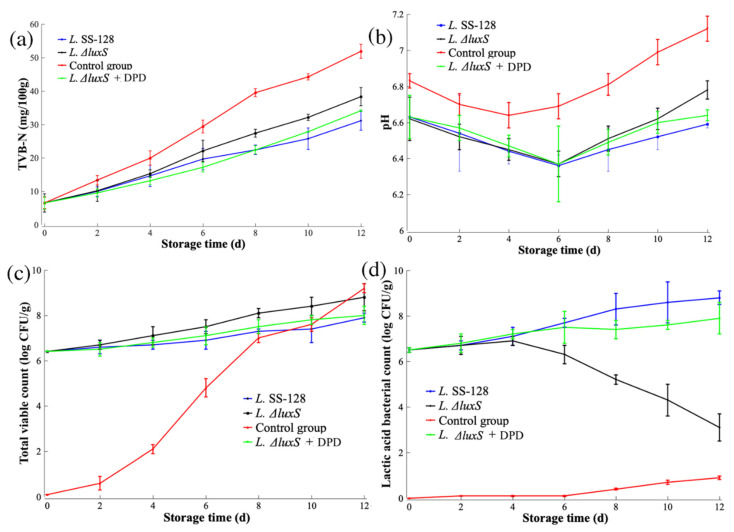
Changes in *Litopenaeus vannamei* during preservation. (**a**) TVB-N; (**b**) pH; (**c**) TVC; (**d**) lactic acid bacterial count.

TVB-N is a critical index referring to the alkaline nitrogen-containing volatile substances, including ammonia and amines, produced by protein decomposition during food spoilage. The higher content of TVB-N is corrected with more destroyed amino acids, especially methionine and tyrosine [34]. TVB-N is used to reflect the freshness of meat and aquatic products, and products with TVB-N values over 30 mg/mL are spoiled [35]. As shown in Figure 5a, in all four groups, the TVB-N value continued to increase during the whole storage period. The TVB-N value of the control group reached 39.54 ± 1.24 mg/mL at 8 d, exceeding the limits of fresh products. The effects of spoilage microorganisms and enzymes are two major causes of the increase in TVB-N. The addition of *L. plantarum* SS-128 significantly slowed the increasing rate of the TVB-N value (*p* < 0.05). At 12 d, the TVB-N value of Group *L.* SS-128 was 27.98 ± 0.21 mg/mL, indicating the acceptable status of *Litopenaeus vannamei.* These results indicated that the participation of *L. plantarum* SS-128 successfully extended the shelf life of *Litopenaeus vannamei* for at least 4 days. There has been some previous research with similar findings. Boulares et al. pointed out that the addition of a lactic acid bacteria mixture could reduce the increasing rate of TVB-N in refrigerated and vacuum-packed sea bass (*Dicentrarchus labrax*) fillets [36]. Kuley et al. proved that fermented sardines inoculated with *Lactobacillus plantarum* FI8595 stored at 3 ± 1 °C retained a TVB-N value below 35 mg/100 g until 7 weeks [37].

Compared to the *L.* SS-128 group, the *L.ΔluxS* group exhibited a significantly higher rate of increase in TVB-N (*p* < 0.05), proving that the luxS gene played a vital role in the storage of *Litopenaeus vannamei*. The addition of DPD to *L.ΔluxS* (Group *L.* SS-128 + DPD) effectively offset the TVB-N increase, indicating that the AI-2/LuxS QS system effectively inhibited the spoilage of *Litopenaeus vannamei*. Li et al. showed that the participation of the AI-2/LuxS QS system in lactic acid bacteria LAB-1 prolonged the shelf life of shrimp [19]. Lee et al. proved that the extracellular vesicles from LAB could exhibit antibacterial activity against *Shewanella putrefaciens*, markedly prolonging the shelf life of refrigerated tuna fish by inhibiting the total volatile base nitrogen (TVBN), oxidation reaction, peroxide value (PV), malondialdehyde (MDA), and bacterial levels [33]. The TVB-N value of Group *L.ΔluxS* at 12 d was 38.34 ± 2.73 mg/mL, which was significantly lower than that of the control group (51.93 ± 2.13 mg/mL, *p* < 0.05), proving that, in addition to the AI-2/LuxS QS system, there might be other mechanisms for the preservation effect of *Litopenaeus vannamei* in both *L. plantarum* SS-128 and *L. plantarum ΔluxS.*

#### 3.4.2. pH

The pH of all of the groups showed a slight decrease in the early stage of storage and then continued to rise. As shown in Figure 5b, due to the acidic environment caused by *L. plantarum* SS-128 or *L. plantarum ΔluxS*, the initial pH of the control group was significantly higher (*p* < 0.05) than that of the experimental groups. Wu et al. considered the decrease in pH in the initial stage for the control group to be related to the accumulation of the product of glycolysis, lactic acid [38]. The decreasing pH period for the three experimental groups was longer (approximately 6 days), which might be a cumulative effect for the metabolism of lactic acid bacteria and the inhibition of spoilage. The increasing pH at the late period of storage is attributed to the growth of spoilage bacteria in *Litopenaeus vannamei* and the subsequent formation of ammonia, alkaline autolysis compounds, and other volatile bases [39]. The pH value between the *L.* SS-128 and *L.ΔluxS* groups showed no significant changes during the early storage of *Litopenaeus vannamei;* however, at the end of storage, the pH value of the *L.* SS-128 group was significantly lower than that of the *L.ΔluxS* group (*p* < 0.05), while the pH between the *L.ΔluxS* and *L.* SS-128 +DPD groups was not significant. According to the bacterial community composition analysis, the AI-2/LuxS QS system in *L. plantarum* SS-128 made it the dominant bacteria at the end of storage, which was attributed to the lower pH of *Litopenaeus vannamei* in the *L.* SS-128 group. The slower spoilage of *Litopenaeus vannamei* was also attributed to the formation of alkaline compounds. Li et al. (2019) found similar trends of pH changes during preservation added with lactic acid bacteria [14].

#### 3.4.3. Total Viable Count (TVC)

The TVC changes during the storage of *Litopenaeus vannamei* are shown in Figure 5c. In the control group, at 8 d, the TVC value was 7.01 ± 0.23 log CFU/g, exceeding the limitation for fresh aquatic products [40], which is consistent with the TVB-N results. The TVC value of the three experimental groups was over 6 log CFU/g due to the inoculation of lactic acid bacteria. With the extension of storage time, the TVC value of those groups increased slightly due to the growth of *L. plantarum* SS-*128*/*L. plantarum ΔluxS* and the competing growth of spoilage bacteria. At the end of the storage, the TVC value of Group *L.* SS-128 (7.9 ± 0.19 log CFU/g) was significantly lower (*p* < 0.05) than that of the control group (9.23 ± 0.21 log CFU/g). According to the bacterial community composition, the spoilage bacteria contributed a subfraction of all the bacterial components. Thus, the total spoilage ability of Group *L.* SS-128 was much lower than that of the control group.

#### 3.4.4. Lactic Acid Bacteria Count

The changes in the lactic acid bacteria count during storage can reflect the competing status with other spoilage bacteria. As shown in Figure 5d, the lactic acid bacteria count in Groups *L.* SS-128 and *L.ΔluxS* +DPD exhibited similar increasing trends throughout storage. Comprehensively analyzing the TVC results, *L. plantarum* SS-*128/L. plantarum ΔluxS* accounted for a high proportion of the total bacteria. However, the lactic acid bacteria count in the *L*.ΔluxS group declined at the late stage of storage, corresponding to inhibition by the spoilage bacteria. These results proved that the AI-2/LuxS QS system effectively assisted the growth advantage of *L. plantarum* SS-128 during the storage of *Litopenaeus vannamei*. Without inoculation with *L. plantarum* SS-128 or *L. plantarum ΔluxS*, the lactic acid bacteria were detected in the control group with a slightly increasing trend. At the end of the storage, the lactic acid bacteria count reached 0.91 ± 0.08 log CFU/g. The lactic acid bacteria have been widely reported to exist during the refrigerated storage of meat and aquatic products [41,42], but are merely considered SSO.

### 3.5. Bacterial Community Composition Analysis

**Figure 6:**
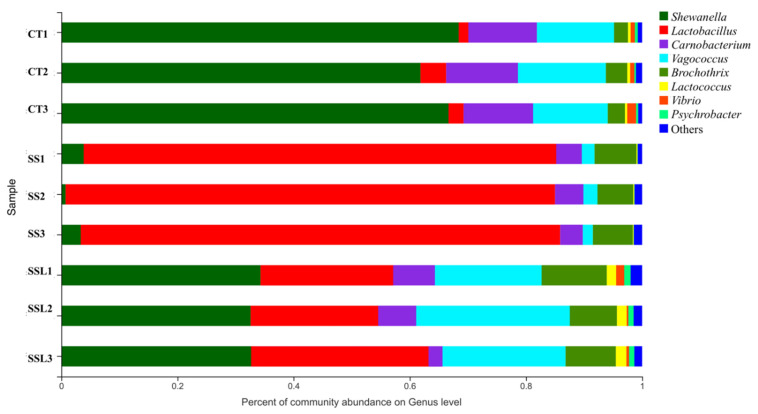
Bacterial community composition.

The bacterial community composition of *Litopenaeus vannamei* incubated with *L. plantarum* SS-128, *luxS* and the control group at the end of storage is shown in Figure 6 (genus level). In the bacterial community composition analysis, the species abundance of each sample was counted, and the community composition was intuitively studied by visualization methods. In the control group, at the end of the storage, *Shewanella* occupied the highest composition of the bacteria (68.42%, 61.84%, 66.67%), followed by *Carnobacterium* (11.80%, 12.36%, 11.97%), and *Vagococcus* (13.29%, 15.14%, 12.86%). Shewanella has been reported to be one of the SSOs with high spoilage potential in *Litopenaeus vannamei* [43] and other aquatic products [6,44]. The abundance of *Shewanella* in the control group during storage indicated the spoilage of *Litopenaeus vannamei*. *Carnobacterium* and *Vagococcus* were also detected during the spoilage of aquatic products [45,46,47].

In the community composition for the group incubated with *L. plantarum* SS-128, *L. plantarum* SS-128 occupied the highest proportion (81.36%, 84.26%, 82.54%), while the growth of most of the major spoilage microorganisms was inhibited to varying degrees. The proportion of *Shewanella*, *Carnobacterium*, and *Vagococcus* was significantly lower than that in the control group, which is evidence that the existence of *L. plantarum* SS-128 suppressed the growth of spoilage microorganisms in *Litopenaeus vannamei* during storage. The extent of the decline in *Shewanella* was the highest, with final proportions in the three parallels of 3.85%, 0.73%, and 3.35%, respectively. The decrease in the spoilage microorganisms was a critical reason for the slower spoilage of *Litopenaeus vannamei* in Group *L.*SS-128. Except for the decreased proportion of the spoilage microorganisms, the kinds of spoilage microorganisms in Group *L.* SS-128 also declined. Vibrio and *Psychrobacter* were barely detected in Group *L.* SS-128, while both were detected in the control group (Vibrio: 0.72%, 0.72%, 1.53%; *Psychrobacter*: 0.50%, 0.28%, 0.50%) and *luxS*-mutant group (Vibrio: 1.31%, 0.29%, 0.47%; *Psychrobacter*: 1.11%, 0.90%, 0.90%). The study by Vinay et al. (2022) showed that *Proteobacteria* is of the most abundant in all of the developmental stages [48]. *Actinobacteria*, *Proteobacteria*, and *Bacteroidota* are reported to be the largest relative abundance of bacterial phyla of Pacific White shrimp *Penaeus vannamei* during an outbreak of white feces disease [49]. *Firmicutes*, *Proteobacteria*, *Desulfobacterota*, *Actinobacteria* and *Bacteroidota* were identified as the five main bacterial phyla in low-salt shrimp paste [50].

The inhibition of spoilage microorganisms by lactic acid bacteria is commonly considered for the following reasons: QS; bacteriocins; organic acids; and peroxide. The *luxS*-mutant group exhibited a lower proportion of *Shewanella* compared to the control group (34.29%, 32.59%, 32.77%), while the content was higher than Group *L.* SS-128, proving that the AI-2/LuxS QS system played an essential role in the storage of *Litopenaeus vannamei*. Without the luxS gene, the *luxS*-mutant group exhibited a better preservative effect than the control group, indicating the diverse effect of lactic acid bacteria in the preservation of *Litopenaeus vannamei*.

## 4. Conclusions

The effect of *L. plantarum* SS-128 on the storage of *Litopenaeus vannamei* was explored in this study through both myofibril simulation and practical application, and the role of the luxS gene was specifically evaluated. The existence of the *luxS* gene can induce AI-2/luxS QS, regulating the growth and metabolism of *S. baltica* A02 in the coculture system. The protein degradation in the myofibril simulation system was significantly inhibited with the addition of *L. plantarum*, proving the inhibitory effect of *L. plantarum* against *S. baltica* A02. During the refrigerated storage of *Litopenaeus vannamei*, the AI-2/LuxS QS system played a critical role, prolonging its shelf life for 4–6 d. The quality of the shrimp in Group *L*. SS-128 was better maintained, exhibited by a lower growth rate for TVB-N and TVC. This study provides an effective biocontrol method for the preservation of aquatic products.

## Data Availability

The data presented in this study are available on request from corresponding author.
